# Integrating optical and electrical sensing with machine learning for advanced particle characterization

**DOI:** 10.1007/s10544-024-00707-0

**Published:** 2024-05-23

**Authors:** Mahtab Kokabi, Muhammad Tayyab, Gulam M. Rather, Arastou Pournadali Khamseh, Daniel Cheng, Edward P. DeMauro, Mehdi Javanmard

**Affiliations:** 1 Department of Electrical and Computer Engineering, Rutgers University, Piscataway, NJ 08854 USA; 2 Rutgers Cancer Institute of New Jersey, Rutgers University, New Brunswick, NJ 08901 USA; 3 Department of Mechanical and Aerospace Engineering, Rutgers University, Piscataway, NJ 08854 USA

**Keywords:** Biosensors, Impedance cytometry, Particle classification, High speed camera, Machine learning, Microfluidic chip

## Abstract

Particle classification plays a crucial role in various scientific and technological applications, such as differentiating between bacteria and viruses in healthcare applications or identifying and classifying cancer cells. This technique requires accurate and efficient analysis of particle properties. In this study, we investigated the integration of electrical and optical features through a multimodal approach for particle classification. Machine learning classifier algorithms were applied to evaluate the impact of combining these measurements. Our results demonstrate the superiority of the multimodal approach over analyzing electrical or optical features independently. We achieved an average test accuracy of 94.9% by integrating both modalities, compared to 66.4% for electrical features alone and 90.7% for optical features alone. This highlights the complementary nature of electrical and optical information and its potential for enhancing classification performance. By leveraging electrical sensing and optical imaging techniques, our multimodal approach provides deeper insights into particle properties and offers a more comprehensive understanding of complex biological systems.

**Graphical abstract:**

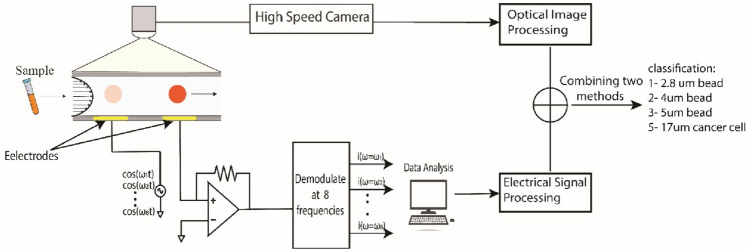

## Introduction

Particle classification plays a pivotal role in various applications, particularly within the healthcare field. One of the widely used techniques for particle classification is flow cytometry (Shvalov et al. 1999; Godavarti et al. 1996). This method enables the analysis and classification of individual cells based on their distinct characteristics such as shape and size. It allows researchers to examine individual cells or particles in a sample, measure their physical and chemical properties, and sort them into different populations based on these characteristics. Moreover, particle classification allows for accurate diagnoses and effective treatment strategies. By analyzing the distinct characteristics of particles, such as shape, size, and surface markers, healthcare professionals can gain valuable insights into the nature of diseases and make informed decisions about patient care. Additionally, particle classification is crucial for infectious disease management. By accurately identifying and differentiating between bacteria and viruses, healthcare professionals can determine appropriate treatment options (Alhadrami 2018).

Microfluidics refers to the manipulation and control of fluids and particles at extremely small scales, typically on the micron and sub-micron levels. This field has experienced rapid growth due to its potential to revolutionize laboratory techniques by reducing sample volumes, enabling faster and more sensitive reactions, separations, and detections at a fraction of the usual time and cost. Microfluidics has the potential to be used in many different areas including high-throughput drug screening (Bachal et al. 2023; Gerlach et al. 2002), analysis and manipulation of single cells or molecules (Kokabi et al. 2023a), drug delivery and advanced therapeutics (Gholizadeh et al. 2022), bio-sensing (Raji et al. 2022; Meng et al. 2024), and point-of-care diagnostics (Yeo et al. 2011; Lin et al. 2023a, b; Javanmard et al. 2023; Ahuja et al. 2019; Kokabi et al. 2023b; Meng et al. 2022). Microfluidic single-cell analysis systems necessitate technological solutions for tasks such as counting, trapping, focusing, separating, sorting, characterizing, and identifying individual cells (Sun et al. 2010; Chao and Ros 2008; Brown and Audet 2008). Through the integration of these technological solutions, microfluidic single-cell analysis systems enable researchers to conduct thorough investigations, unveil cellular diversity, and gain essential insights into biological processes at the individual cell level. Impedance stands out as a pivotal parameter employed in microfluidics for both cell identification and quantification (Raji et al. 2022; Kokabi et al. 2023a; Ahuja et al. 2019; Song et al. 2021; Daguerre et al. 2020). When a cell traverses the electrodes within a microfluidic channel, it induces a change in impedance (Kokabi et al. 2023a). The resulting output signal reflects the cell’s intrinsic properties, including its size, conductivity, and permittivity (Sui et al. 2021). These properties contribute to the observed modulation in impedance. By analyzing the impedance signal, researchers can derive information about the physical characteristics of the cells under study. Impedance-based measurements offer significant advantages in microfluidic single-cell analysis. They provide a non-invasive and label-free approach that allows real-time monitoring of cells (D’Orazio et al. 2021; Kemna et al. 2013; Pfützner et al. 2004). This eliminates the need for additional labeling or sample preparation steps, enabling rapid and continuous analysis.

In addition to electrical detection methods such as impedance cytometry, optical detection plays a prominent role in cell analysis (Raji et al. 2022; Zheng et al. 2010; García-Hernández et al. 2023; Chiu et al. 2015). Optical detection involves the utilization of optical techniques and instruments for detection, classification, and stratification of cells. Two widely employed approaches in optical detection are image-based detection and optical flow cytometry (Raji et al. 2022; Tuchin 2011, 2014). Image-based detection leverages the power of machine learning (ML) techniques to extract relevant information and features from images or videos of cells (Raji et al. 2022; Lin et al. 2023a, b; Cui et al. 2020; Pirsaheb et al. 2019). This visual data needs processing to identify and quantify cells accurately. ML algorithms are well-known for their ability to make accurate predictions on extensive datasets, significantly enhance the analysis of image data. By integrating ML algorithms, manual data processing steps can be minimized, resulting in reduced processing time and the elimination of potential human errors (Ventura et al. 2021; Li et al. 1999; Rosten et al. 2006).

Optical detection offers distinctive advantages, including immunity to electromagnetic interference, corrosion resistance, and high sensitivity (Chen and Wang 2020; Ramsden 1997; Lechuga 2005). Optical sensing uses a different method to examine distinct characteristics of particles. As a result, combining electrical and optical sensing techniques in a multimodal approach has the potential to improve recognition accuracy compared to using each technique separately (D’Orazio et al. 2021; Sabri et al. 2013).

**Fig. 1:**
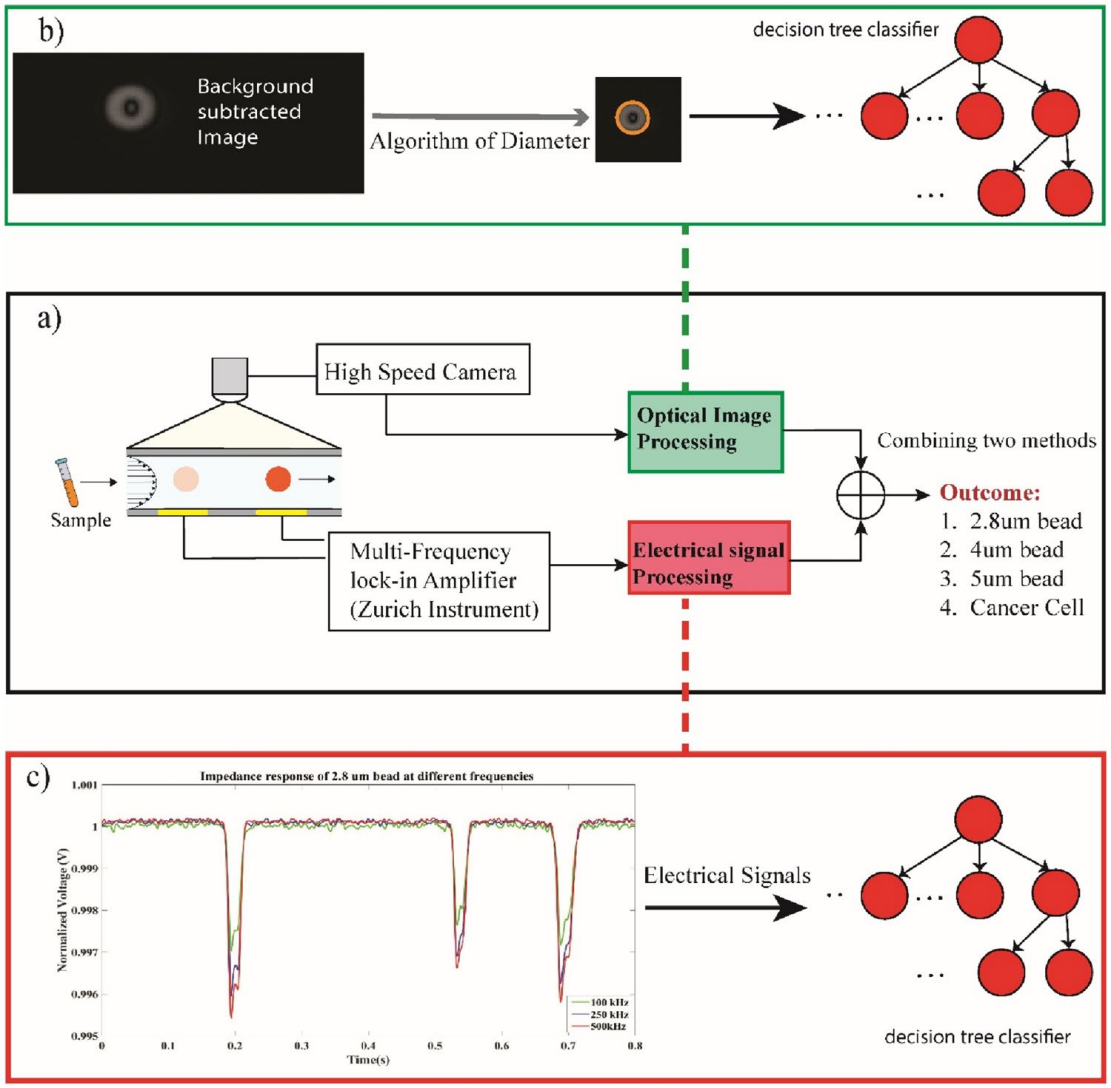
Model overview of system. **a** A multi-frequency lock-in amplifier and a high-speed camera are connected to a microfluidic channel to enable simultaneous optical and electrical measurements. **b** Image processing flow involves background subtraction and obtaining particle volume as a potential optical feature for classification. **c** Electrical signal processing flow: impedance responses are obtained as a potential electrical feature for classification

To validate this hypothesis, we conducted simultaneous experiments involving paramagnetic beads of varying sizes and breast cancer cells. Figure 1 provides an overview of the system setup. The setup consists of two main parts: an electrical module and an optical module. The optical module incorporates a high-speed camera for capturing particle videos under a microscope. The images obtained from the video are then analyzed, and the volume of particles is obtained as a potential optical feature. The electrical module involves the use of a Zurich Instruments Lock-in Amplifier for recording impedance responses. Impedance responses of all particles and cancer cells are recorded, and the corresponding volume of each particle has been obtained. These two features, including volume obtained from the optical part and impedance obtained from electrical measurements, are then combined to boost the classification accuracy of the system.

For particle classification, we utilized machine learning classifier algorithms on both electrical and optical features, as well as a combined feature set, to assess the impact of integrating these two types of features. To evaluate the proposed model’s performance, we conducted separate experimental series for training and testing purposes. These experiments were meticulously designed to capture potential variability in impedance responses stemming from differences between devices and daily environmental. In addition, we implemented various machine learning classifier algorithms and selected the best classifier algorithm using MATLAB’s classifier framework to understand the effects of combining these feature sets. Detailed information regarding the materials and methods can be found in the next section.

## Materials and methods

### Microfluidic device fabrication

The microfluidic device was fabricated with standard photolithography, electron beam evaporation, and lift-off processing on a 3-inch glass wafer. The fabricated biosensor consists of two layers, the first layer is a microfluidic channel made of polydimethylsiloxane (PDMS), and the second layer is a pair of electrodes. The electrodes are 20 μm in width and the gap between two electrodes are 30 μm. The microfluidic channel was fabricated using soft lithography with a layer of SU-8 patterned on a 3-inch silicon wafer. The process involving spin coating, soft bake, UV exposure, development, and finally hard bake. The width of microfluidic channel is 50 μm, which is large enough in our study to minimize clogging and small enough to obtain sufficient sensitivity during measurements. The microfluidic channel mold fabricated in the cleanroom and the biosensor pattern was transferred from the mold to a PDMS slab by mixing pre-polymer and curing agent with the mass ratio of 10:1. After pouring the mixture onto the channel mold and baking for 30 min at 80 °C, the sensor was peeled off and two holes of 5 mm were punched as the inlet and outlet of the biosensor chip. The PDMS chip was covalently bonded onto the glass electrode chip by treating the two substrates with oxygen plasma.

**Fig. 2:**
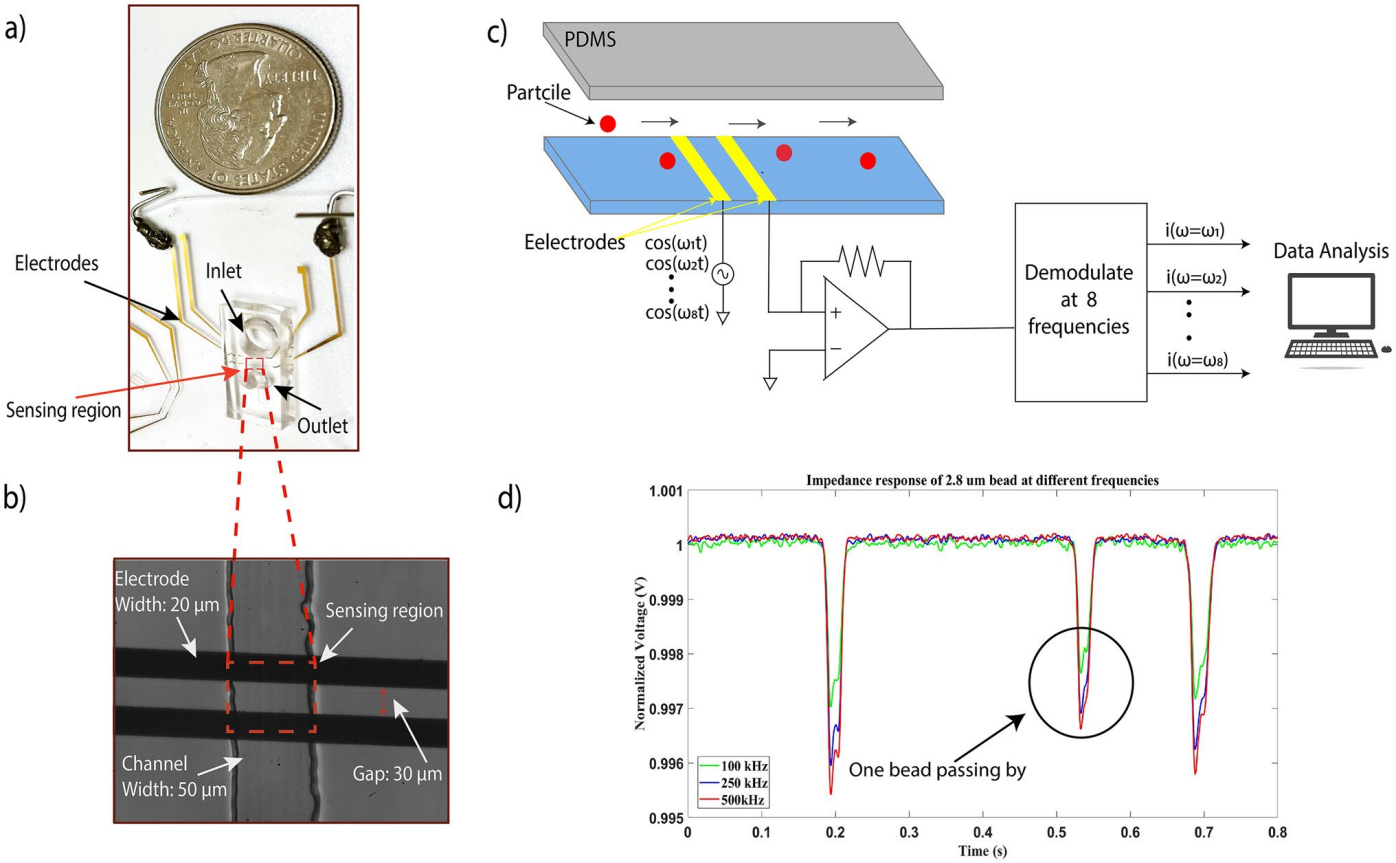
**a** Image of the device in which a soft lithography made PDMS is integrated with electrodes patterned on the fused silicon wafer. **b** The microscope image of channel and electrodes. **c** The schematic diagram of detection is as follows: Particles are injected into the inlet well using a micro pipette. As particles flow through the pore, the impedance changes are captured by the lock-in amplifier at multiple frequencies. The data is then sent to the PC and analyzed in MATLAB. **d** Representative data of bare 2.8 µm paramagnetic beads passing through the sensing region measured at 100 kHz, 250 kHz, and 500 kHz frequencies respectively

The second layer of the biosensor is a pair of electrodes made in the cleanroom with the following process: spin coating, pre-bake, UV exposure, development, and post bake. The process started with spin coating of positive photoresist (AZ 5241E) on the glass wafer. After the spin coating process, the wafer baked for 60 s at 95 °C. Followed by UV exposure and development process, the desired pattern transferred from mask to the wafer. 5 nm adhesive layer of chromium and a 100 nm layer of gold sequentially were deposited on the wafer by using electron beam evaporation. Then, the unwanted pattern was lifted off by placing the wafer in acetone. Figure 2a illustrates the image of the biosensor in which the PDMS channel is integrated on the electrodes. Figure 2b demonstrates the microscopic image of the microfluidic channel and electrodes. Particles are injected into the inlet of the microfluidic sensor, and the changes in impedance are captured by the lock-in amplifier at multiple frequencies. The schematic diagram of detection is illustrated in Fig. 2c. Figure 2d shows the representative multi-frequency time series data of bare paramagnetic beads in a 0.8 s time window. Traces are normalized with respect to the baseline impedance for ease of comparison.

### Sample collection and preparation

In this experiment, three different Magnetic Polystyrene Particles beads of varying sizes were tested, along with one type of breast cancer cell. Table 1 illustrates the particle sizes of the paramagnetic beads and breast cancer cell. Classes 1 to 3 belong to different types of beads, while class 4 belongs to breast cancer cells.

**Table 1:** Nominal size of particles and cells

Class	Particle	Nominal size
1	Magnetic Polystyrene Particles	2.0–2.9 μm
2	Magnetic Polystyrene Particles	4.0–4.5 μm
3	Magnetic Polystyrene Particles	5.0–5.9 μm
4	MDA-MB-231, Breast Cancer Cell	15.0–17.0 μm

The MDA-MB-231 cell line, derived from a pleural effusion of a 51-year-old Caucasian female diagnosed with metastatic mammary adenocarcinoma, is an epithelial human breast cancer cell line. It is widely utilized in medical research laboratories and represents one of the most frequently employed cell lines for breast cancer studies (Palacios Ruiz 2022; Bakhshpour et al. 2019; Karki et al. 2023). MDA-MB-231 cells were cultured in the MEM medium (Minimum Essential Medium) (Life technologies, Grand Island, NY, catalog number 32561-037-500) with 10% FBS (Life Technologies, Grand Island, NY), 1% penicillin / streptomycin (Life Technologies, Grand Island, NY) in an incubator with atmosphere of 5% CO2 and 37 ºC. The cell line was obtained from American Type Culture Collection (ATCC) and was checked for mycoplasma by MycoAlert mycoplasma detection kit (Lonza USA) before starting any experiment (Rather et al. 2018; Shaik et al. 2018). MDA-MB-231 cells were grown in a 100 by 55 mm petri-dish. Upon reaching to 70–80% confluency, growth media was aspirated, and cells were washed with 1X phosphate-buffered saline (PBS) twice and trypsinzied with 1 mL trypsin (0.25%) for 5–10 min in an incubator and collected using 2–3 mL growth media in a 15-mL collection tube. The cells were homogenized with the growth media and cell viability was determined using the Vi-CELL Series Cell Viability Analyzer (Beckman Coulter, Carlsbad, CA) (Kabakov et al. 2021). Cells greater than 95% viability were used for further experiments. Finally, one million viable cells were resuspended in 1 mL of 1X PBS after being washed twice with 1X PBS to remove all the growth media components, before being used for the current study.

### Experimental setup

**Fig. 3:**
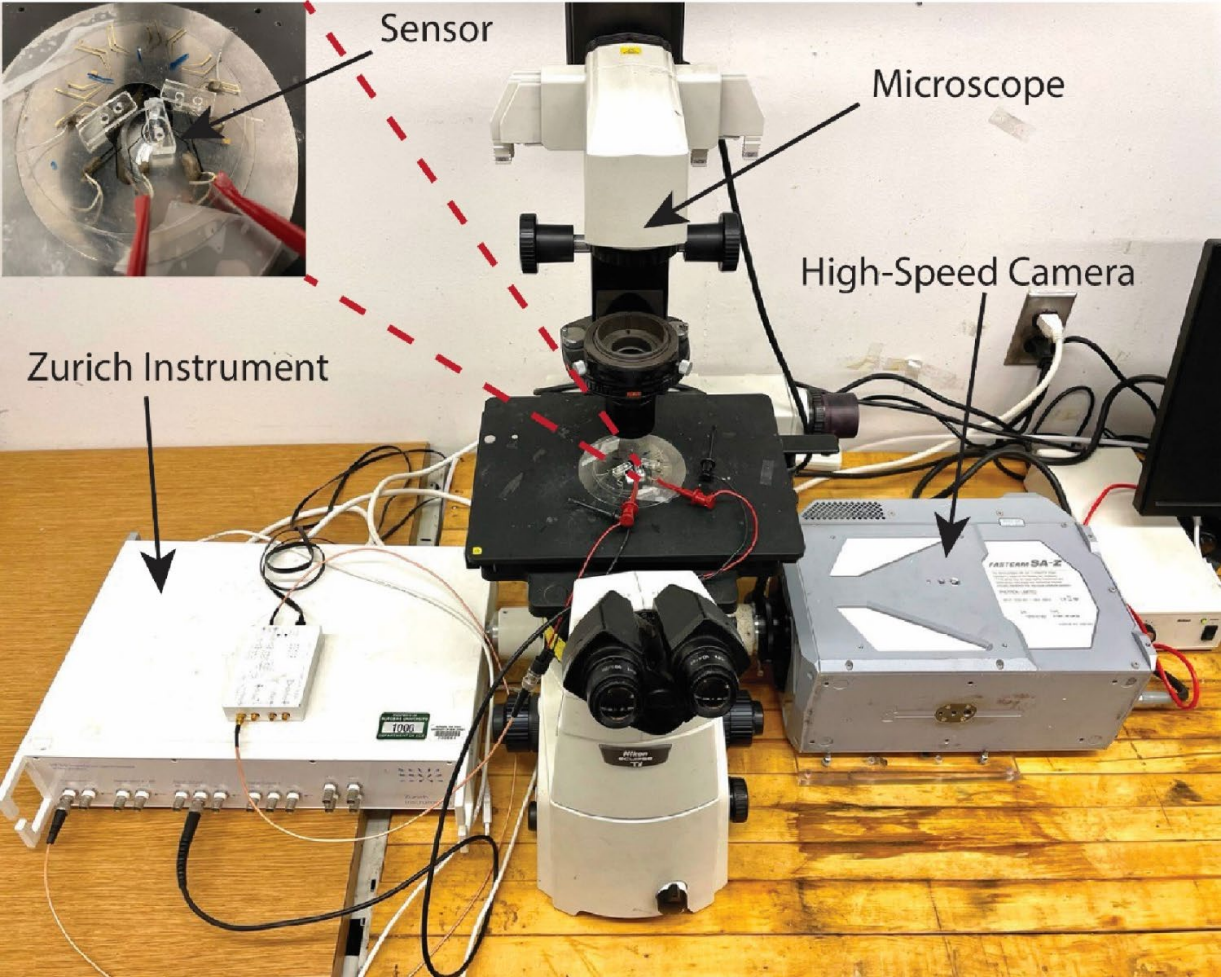
Experimental setup including lock in amplifier (Zurich instrument) and Photron Fast-CAM high-speed camera

 The experimental setup is depicted in Fig. 3. To measure the impedance signals, an HF2IS impedance spectroscope (Zurich Instrument) was utilized. In each individual experiment, either breast cancer cell or a group of beads with the same nominal size was injected into the microfluidic biosensor, and the corresponding impedance signals were recorded. Concurrently, a high-speed camera was connected to a microscope to capture the passage of a flowing particle. The electrical signals and synchronized optical images were processed using machine learning-based classifier algorithms, which provided a probability indicating the likelihood of the particle belonging to a specific bead group or being a breast cancer cell.

To process the acquired electrical signals and synchronized optical images, a machine learning-based classifier algorithms were employed. These algorithms were specifically designed to analyze the impedance signals and the corresponding optical images simultaneously. By leveraging the power of machine learning, the classifiers were able to effectively extract meaningful features and patterns from the data. The ultimate goal of this experimental setup was to develop a reliable classification system. By combining information from both the impedance signals and the optical images, the machine learning-based classifiers assigned a probability indicating the likelihood of a particle belonging to a specific bead group or being a breast cancer cell. This integration of multiple data sources improved the accuracy and robustness of the classification process.

In the subsequent sections, a detailed explanation of the impedance measurement methodology and the image acquisition process will be provided, shedding light on the technical aspects and the significance of each component in the experimental setup.

### Impedance acquisition and analysis

To obtain precise electrical measurements, a multi-frequency lock-in amplifier (Zurich Instruments HF2A, Zurich, Switzerland) was employed in combination with an impedance spectroscope (HF2IS, Zurich Instruments), which operated at a sampling rate of 899 samples per second. Additionally, a trans-impedance amplifier (HF2TA, Zurich Instruments) was utilized to enhance the measurement accuracy. The impedance spectroscope allowed for the characterization of the impedance signals generated by the beads or particles passing through the sensing region. These signals provided valuable information about the electrical properties and interactions between the particles and the microfluidic biosensor.

To establish the electrical connection between the biosensor device and the lock-in amplifier, two wires were bonded to the gold pad of electrodes on the biosensor. This connection enabled the transmission of electrical signals for subsequent analysis. Before initiating the experiments, the bare magnetic beads and cancer cells were suspended in phosphate-buffered saline (PBS) to ensure a suitable environment for their introduction into the microfluidic device. To inject the particles or cells into the biosensor, a pipette pump was utilized. Each set of experiments involved injecting cells or the same type of beads within a specific size range into the sensor. The aim was to evaluate the impedance responses associated with different groups of beads or breast cancer cells, thereby enabling the development of classification algorithms based on distinctive electrical features.

The experimental procedure consisted of several stages. Initially, three experiments were conducted using bare magnetic beads with nominal sizes ranging from 2.0 to 2.9 μm, 4.0 to 4.5 μm, and 5.0 to 5.9 μm, respectively. These experiments aimed to capture the impedance characteristics and electrical signals specific to each bead size range. Subsequently, a similar experiment was performed using MDA-MB-231 breast cancer cells. This allowed for the comparison of impedance responses between the magnetic beads and cancer cells, further enhancing the classification capabilities of the system.

To ensure an adequate dataset for analysis, each experiment was run for a sufficient duration until several hundred peaks in the electrical signals were obtained. This ensured a robust representation of the impedance responses, providing a comprehensive basis for subsequent analysis and classification. To comprehensively evaluate the impedance response, measurements were simultaneously taken at eight different frequencies: 100 kHz, 250 kHz, 500 kHz, 750 kHz, 1 MHz, 1.25 MHz, 1.5 MHz, and 1.75 MHz. By employing these measurement techniques and carefully selecting the frequencies, the experimental setup enabled the acquisition of highly detailed impedance responses for different bead groups and breast cancer cells. The impedance responses allowed for a comprehensive exploration of the electrical behavior of the particles, facilitating the subsequent development of robust classification algorithms.

### Image acquisition and analysis

**Fig. 4:**
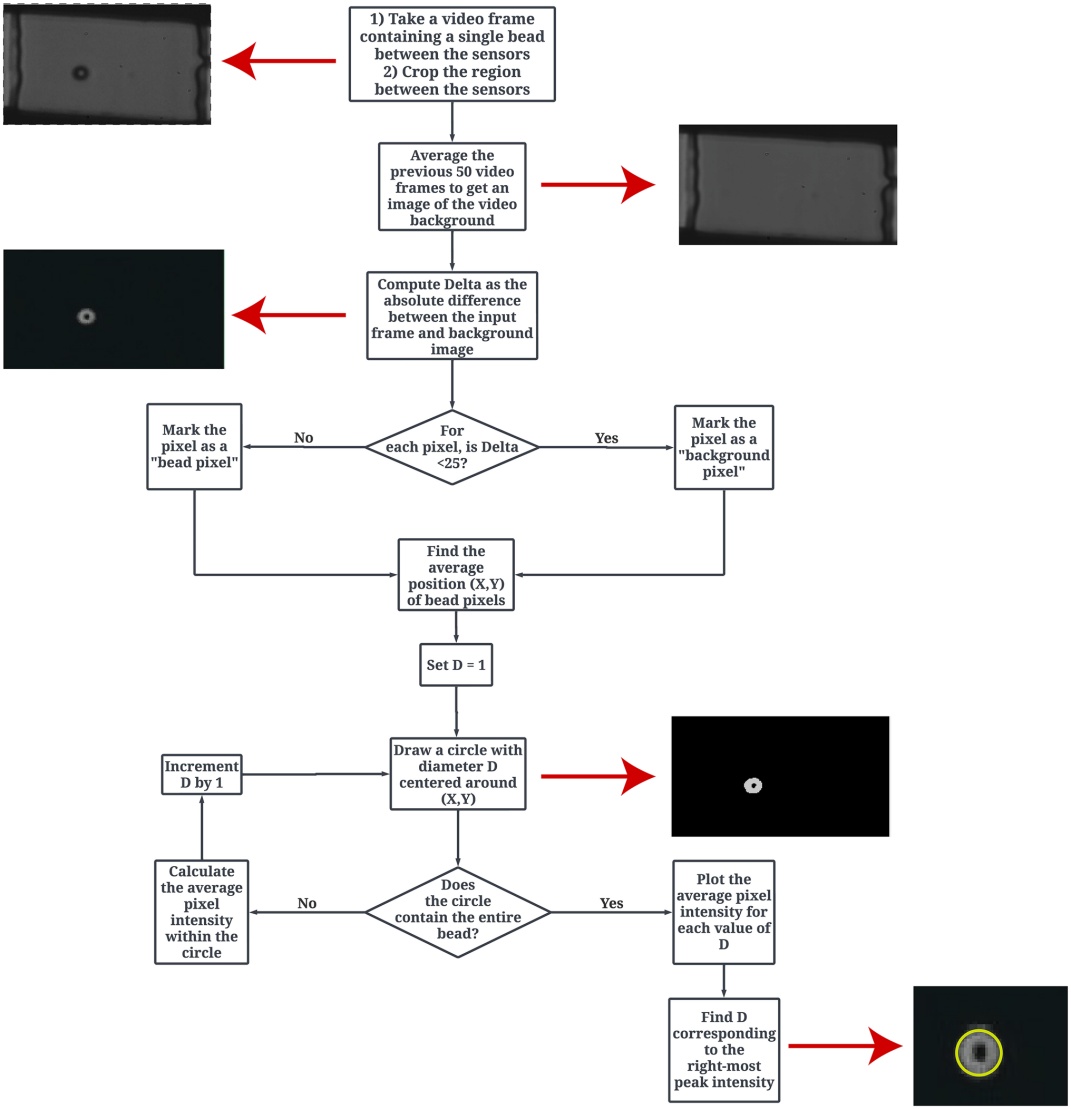
Diameter algorithm flowchart

 The injected samples passed through the microfluidic channel, and the electrical measurements were collected according to the previous section. In addition to impedance signals, a high-speed camera was used to simultaneously perform particle visualization. This allows cross-examination between the collected impedance data and optical data. Image recording was performed using a Photron FAST-CAM SA-Z high-speed monochrome camera connected to a microscope. The camera settings were set to 125 frame per seconds (fps) and 63.75 µs exposure time, which is fast enough in our study to capture beads and cells. The recorded video obtained by high-speed camera was extracted to its frames. The optical image acquisition was synchronized with the impedance measurements. The next step is to find the particle volume and its correlation with the impedance measurement. In order to find a particle volume, a custom written MATLAB code is used to find the particle diameter. Figure 4 illustrates the algorithm that was used to find the particle’s diameter.

The diameter algorithm processing flow is as follows: A ROI (Region of Interest) is defined where the beads or cells are located between two electrodes. For video frames containing a single isolated bead, a MATLAB algorithm was used to automatically find the diameter of the bead. First, an image of the blank channel was created by calculating the average of the 50 previous frames in the video. The absolute difference is taken between the frame containing the bead and the blank image, forming a new “difference image”, which eliminates the bead’s surroundings from the image. The bead can be identified by the ring of white pixels, in contrast to the background pixels which have their intensity eliminated to nearly 0 and appear black.

To calculate the diameter of the bead, it is necessary to locate the position of the bead’s center. This process involves scanning through the difference image and distinguishing between the pixels that belong to the bead and those that correspond to the background. Since the bead pixels appear brighter in the difference image, an intensity threshold is set. Any pixels that exceed this threshold are marked as bead pixel. To determine the position of the bead, the x and y positions of all marked bead pixels are averaged. Once the center of the bead is found, the diameter of the bead can be determined. By plotting the average pixel intensity against the distance from the center of the bead, the dimensions of the bead can be identified. This intensity profile enables the calculation of the bead’s diameter.

Once the diameter is obtained, the volume (V) of the particle can be determined using the formula for the volume of a sphere: $V=\frac{4}{3}\pi {r}^{3}$, where r represents the radius of the bead (which is half of the diameter). This methodology combines image analysis techniques with intensity profiles to accurately determine the diameter and volume of the bead. By leveraging these calculations, valuable insights into the physical characteristics of the particles can be obtained for further analysis and classification.

### Machine learning analysis

Optical images were synchronized with impedance signals, and a MATLAB algorithm was employed to extract the diameter of each particle from the optical images. In this study, a novel multi-modal framework is introduced, wherein impedance signals and optical measurements are combined as input features for a machine learning classifier model. This framework aims to classify particles, specifically beads or cancer cells, within a microfluidic chip by integrating electrical sensing features and optical imaging. The input features of the machine learning classifier consist of the particle volume obtained from the optical images and the impedance signals obtained from the Zurich instrument. The output of the machine learning model provides the probability of a particle belonging to different bead groups or being a cancer cell.

For the development of the classification model, we utilized the MATLAB Classification Learner application toolbox. This toolbox facilitated the training of the model using the provided input features. To evaluate the proposed model’s performance, we undertook a separate series of experiments to create a test dataset, which is approximately one-third the size of the training dataset. This approach ensures a robust assessment of the model against genuinely unseen data. These experiments were designed to account for potential variations in impedance responses caused by device-to-device differences and daily environmental fluctuations. Subsequently, the performance of the multi-modal classifier was compared to that of the individual electrical and optical measurement features. By intentionally introducing such variability, our aim was to simulate the diverse conditions under which the classifier would operate in practical settings, ensuring that our evaluation reflects its performance in the face of real-world complexities. By implementing these additional experiments and analyses, we have significantly enhanced the representativeness and diversity of our training and test datasets. These efforts ensure that our model’s performance is not merely a reflection of overfitting to a limited range of data but is indicative of its true capability to generalize across a wider spectrum of particle types and conditions encountered in practical applications.

To select the most suitable algorithm for the multi-modal framework, we assessed 25 available algorithms within the MATLAB classification learner application. The fine tree machine learning algorithm demonstrated the highest accuracy values and was chosen for this study. Fine tree algorithm is a subset of decision tree algorithms and is employed for both regression and classification tasks. It utilizes up to 100 decision rules (i.e., decision trees) to achieve precise classification of the data (Kokabi et al. 2020), making it well-suited for our purposes. The fine tree algorithm distinguishes itself through its capacity to handle complex datasets by employing multiple layers of decision trees, which intricately segment the data based on a series of binary questions. This segmentation process allows for the handling of nonlinear relationships and interactions among variables with remarkable finesse. The algorithm’s strength lies in its simplicity and interpretability, as each decision node in the tree represents a clear and logical decision point, making the reasoning behind each classification or regression decision transparent.

We implemented 5-fold cross-validation to combat overfitting, dividing the training dataset into five subsets and iteratively training the model on four subsets while using the fifth for validation. This process, repeated five times with each subset serving as the validation set once, allowed for a comprehensive evaluation of the model’s performance on unseen data. By implementing the fine tree algorithm and combining impedance signals and optical measurements as input features, our multi-modal framework offers an effective approach for particle classification in the microfluidic chip. The utilization of machine learning techniques enhances the accuracy and robustness of the classification process, contributing to improved particle characterization and identification.

## Results and discussion

### Optical measurement validation

In this study, each group of beads and cancer cells was appropriately labeled (as shown in Table 1) to distinguish between different categories. Class 1, 2, and 3 corresponded to average bead sizes of 2 μm, 4 μm, and 5 μm, respectively. Class 4 represented the group of cancer cells. In each individual experiment, only one type of beads or cells passed through a PDMS microfluidic channel. Impedance cytometry techniques were then employed to detect impedance differences between beads of different sizes and cells. The impedance response was measured at 8 different frequencies using a multi-frequency lock-in amplifier (Zurich Instruments HF2A, Zurich, Switzerland). Concurrently, optical images were captured using a high-speed camera.

**Fig. 5:**
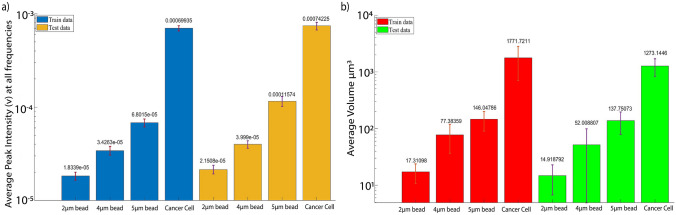
**a** Average peak intensity (PI) for each individual particle across all frequencies in both the training and test dataset **b** Average volume in both the training and test dataset

Figure 5a demonstrates the average impedance across all 8 frequencies for each individual particle category in both the training and test datasets. As depicted, there is a noticeable increase in impedance with larger particle sizes. Figure 5b illustrates the average volume for each particle category. In situations involving the aggregation of beads or cancer cells, the volume of each individual particle is calculated, and the final volume is determined by summing the volumes of all the individual particles.

**Fig. 6:**
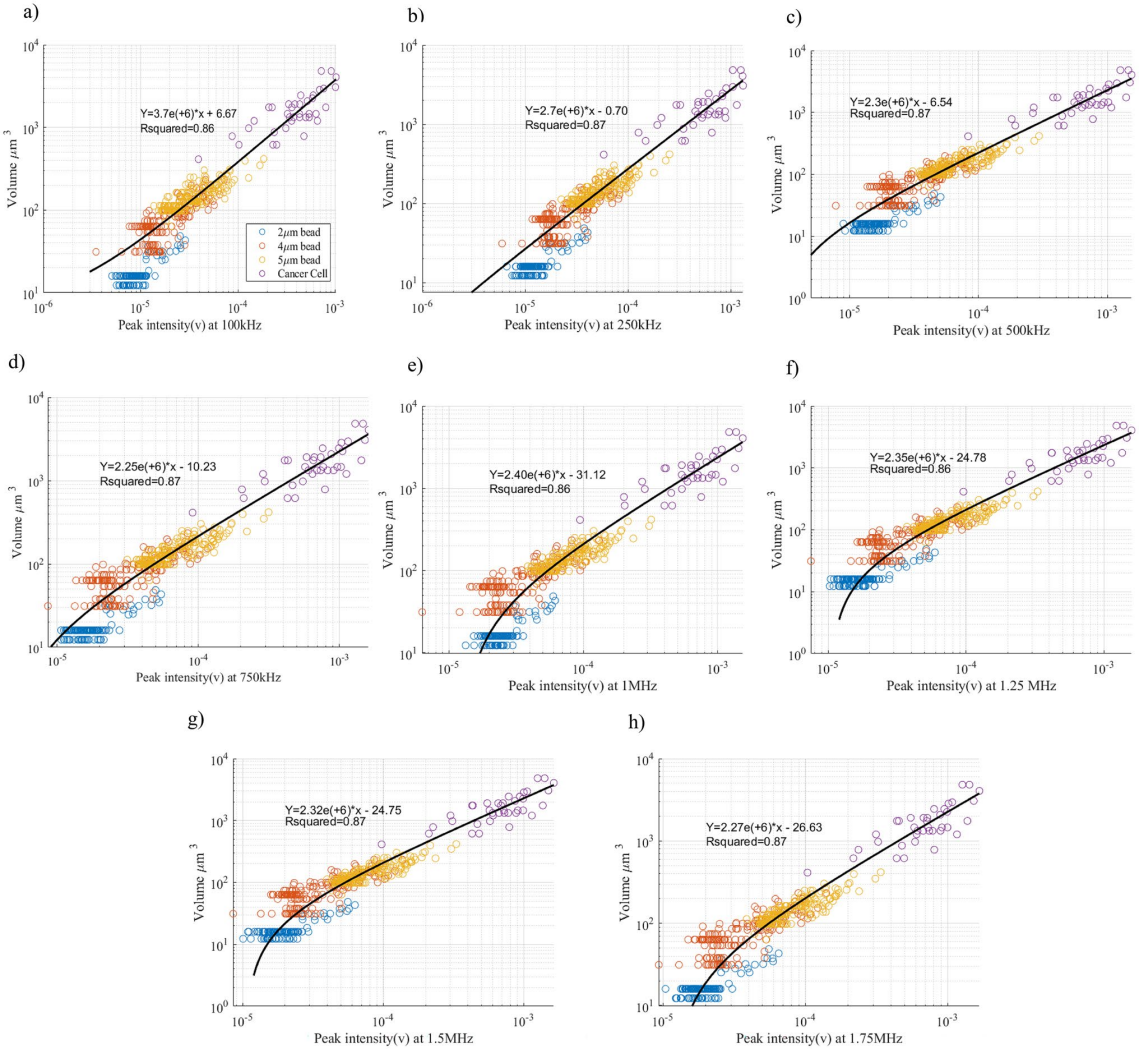
Scatter plots illustrating the relationship between volume and impedance. A linear regression line has been applied to the data at 8 different frequencies including: **a** 100 KHz **b** 250 KHz **c** 500 KHz **d** 750 KHz **e** 1 MHZ **f** 1.25 MHZ **g** 1.5 MHZ **h** 1.75 MHZ

 To validate the accuracy of the optical measurements obtained from the high-speed camera, a linear regression model was employed for data analysis. Figure 6 illustrates the linear relationship between the particle volume derived from the high-speed camera images and the impedance measurements obtained from the Zurich instrument, for each of the 8 frequencies. For better visualization of the linear regression, both impedance and volume are depicted in a logarithmic scale. As depicted in Fig. 6, a positive correlation exists between the particle volume and its impedance. This relationship indicates that as particle volume increases, impedance also increases.

Several factors contribute to the impedance of a material, including the size and shape of its constituent particles. With an increase in particle size, impedance tends to rise. This phenomenon arises due to the larger surface area and modified surface-to-volume ratio of larger particles. These alterations influence the interaction of the material with the AC signal traversing through it, leading to variations in impedance.

Figure 6 provides visual evidence of the linear regression relationship between the particle volume and the peak intensity (impedance), with an average R-squared value of 86%. This high R-squared value indicates a relatively strong correlation between the two variables. The linear regression analysis confirms the reliability and consistency of the optical measurements in capturing information about the particle volume, which correlates with the impedance response. Table 2 represents the R-squared values of Volume vs. Peak Intensity for different frequencies. It also shows the R-squared values of Volume, Peak Intensity, and Velocity for various frequencies. The data indicates that particle velocity is not considered as a potentially informative parameter, as the R-squared value remains unchanged. The reason for this is that the flow of particles in the channel is not dependent on particle size in our case, due to potential variations in channel clogging. In the multimodal classification, we only applied volume and impedance measurements as the two potential informative features for classification.

**Table 2:** R-squared values of volume (VOL) vs. peak intensity (PI), as well as VOL and PI vs. velocity (VEL), for both the train and test datasets

	Rsquared VOL vs. PI		Rsquared VOL and PI vs. VEL	
Frequency	Train	Test	Train	Test
100 KHz	0.86	0.89	0.86	0.89
250 KHz	0.87	0.88	0.87	0.88
500 KHz	0.87	0.87	0.87	0.87
750 KHz	0.87	0.86	0.87	0.86
1 MHz	0.86	0.86	0.86	0.86
1.25 MHz	0.86	0.91	0.86	0.91
1.5 MHz	0.87	0.92	0.87	0.92
1.75 MHz	0.87	0.92	0.87	0.92

**Fig. 7:**
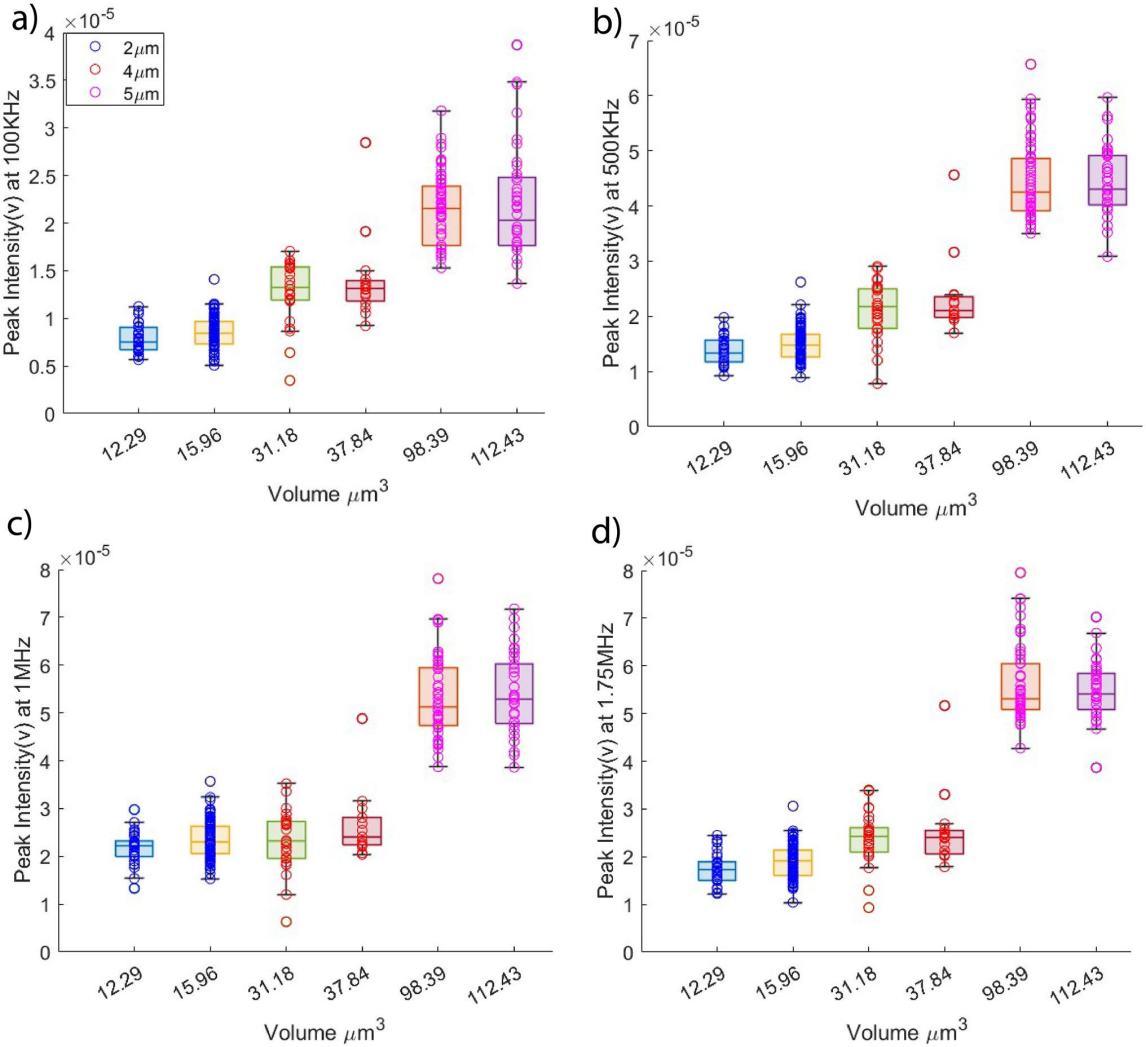
Box plots of peak intensity vs volume at different representative frequencies including **a **100KHZ, **b** 500 KHz, **c** 1MHZ, and **d** 1.75MHZ

 Figure 7 illustrates box plots depicting different representative volumes. Notably, each representative volume is associated with multiple peak intensity values. This occurrence is attributed to the low resolution of the high-speed camera utilized. Specifically, each pixel within the captured image corresponds to approximately 0.2 μm due to constraints inherent to the high-speed camera’s capabilities. Given these circumstances, the combination of volume and peak intensity as classification features becomes a compelling strategy. By leveraging the complementary information provided by these two features, the classification model can potentially overcome the limitations of individual measurements and enhance the accuracy of particle categorization.

### Multi-modal classifier

In this section, we will be providing machine learning algorithms with both electrical and optical features to examine their respective effects. By incorporating both types of features into the analysis, we aim to gain a comprehensive understanding of their individual impacts and potential synergies. Through this approach, we can assess how electrical and optical characteristics influence the performance and outcomes of the machine learning algorithms employed. This perspective will enable us to identify key insights and determine the optimal combination of features for our purposes. To evaluate the performance of a machine learning model, the following metrics are commonly used: accuracy (ACC), true positive rate (TPR), true negative rate (TNR), false negative rate (FNR), and false positive rate (FPR). These measures are computed using the following forms:

1 $$\text{A}\text{c}\text{c}\text{u}\text{r}\text{a}\text{c}\text{y}\; \left(\text{A}\text{C}\text{C}\right)=\frac{\text{T}\text{P}+\text{T}\text{N}}{\text{T}\text{N}+\text{T}\text{P}+\text{F}\text{N}+\text{F}\text{P}}$$

2 $$\text{S}\text{e}\text{n}\text{s}\text{i}\text{t}\text{i}\text{v}\text{i}\text{t}\text{y}\; \left(\text{T}\text{R}\text{P}\right)=\frac{\text{T}\text{P}}{\text{T}\text{P}+\text{F}\text{N}}$$

3 $$\text{S}\text{p}\text{e}\text{c}\text{i}\text{f}\text{i}\text{c}\text{i}\text{t}\text{y}\; \left(\text{T}\text{N}\text{R}\right)=\frac{\text{T}\text{N}}{\text{T}\text{N}+\text{F}\text{P}}$$

4 $$\text{F}\text{a}\text{l}\text{l}\text{o}\text{u}\text{t}\; \left(\text{F}\text{P}\text{R}\right)=\frac{\text{F}\text{P}}{\text{T}\text{N}+\text{F}\text{P}}$$

5 $$\text{False Negative Rate (FNR)}=\frac{\text{F}\text{N}}{\text{T}\text{P}+\text{F}\text{N}}$$

where, TPs [FPs] refer to the number of correct [incorrect] predictions of outcomes to be in considered output class, whereas TNs [FNs] refer to the number of correct [incorrect] predictions of outcomes to be in any other output classes (Kokabi et al. 2020). In the preceding section, we conducted an evaluation of 25 available algorithms using the MATLAB Classification Learner application. Among these algorithms, the fine tree machine learning algorithm exhibited the highest accuracy values and was selected for this study. Our objective was to assess the algorithm’s performance in classifying different particle classes. Specifically, we employed the fine tree algorithm to classify each particle class based on electrical, optical, and a combination of electrical and optical features.

**Fig. 8:**
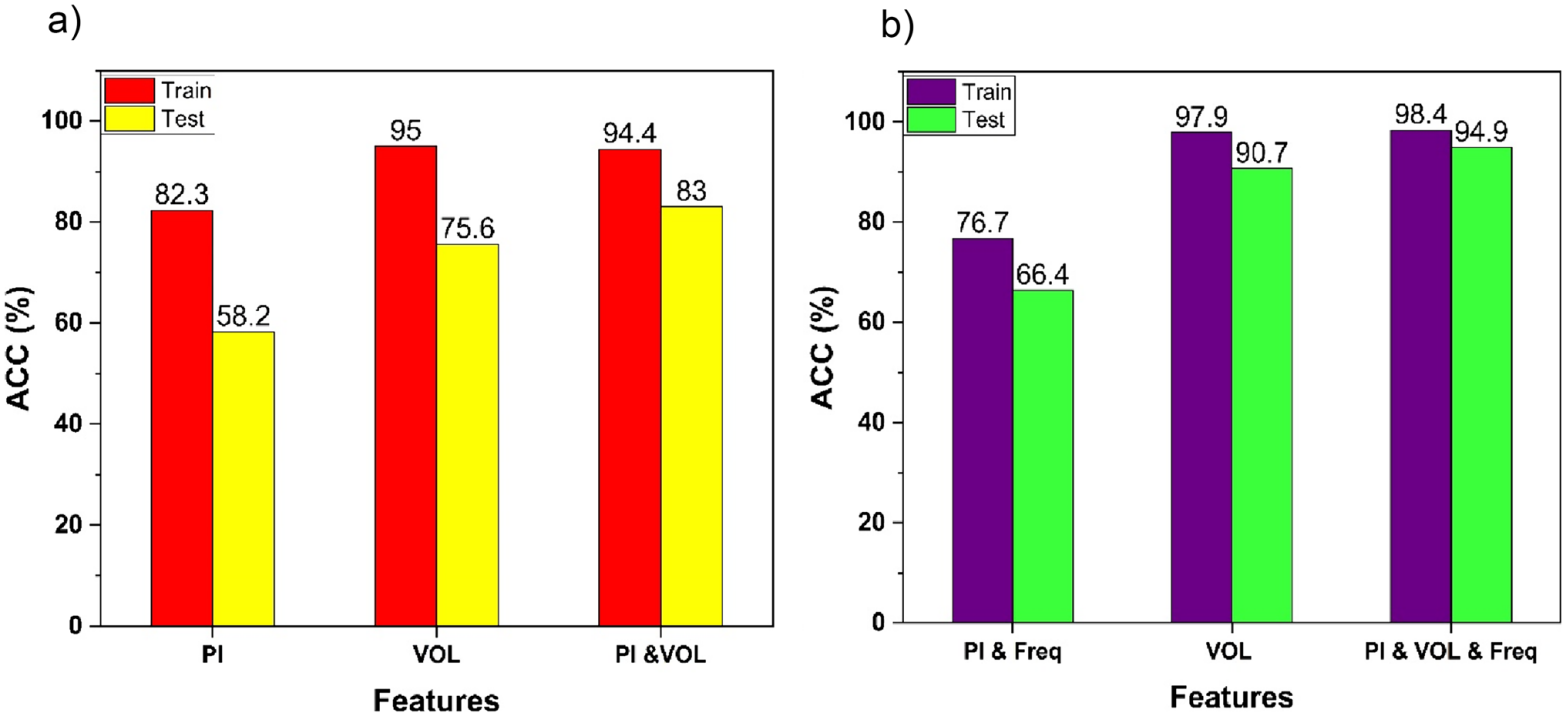
**a** Train and test accuracy on electrical (PI), optical (VOL) and integration of both features (PI and VOL) at 500KHz frequency **b** Train and test accuracy on electrical (PI), optical (VOL) and integration of both features (PI and VOL) with combination of all 8 frequencies

In Fig. 8a, the classification accuracy is represented at a single representative frequency (500 kHz). With the chosen frequency of 500 kHz, three different categories of features are fed to the machine learning model, including peak intensity (PI), volume (VOL), and a combination of PI and VOL. Notably, a noteworthy improvement of approximately 17% is observed when employing the optical feature instead of the electrical feature. This emphasizes the effectiveness of the optical feature in accurately categorizing particles. To showcase the capabilities of both optical and electrical features, a machine learning algorithm was applied to classify particles using both sets of features. Remarkably, this approach yielded an additional improvement of approximately 8\% in accurately classifying particles on the test dataset. The findings underscore the advantage of integrating optical features in particle classification, leading to enhanced accuracy compared to relying solely on electrical or optical features.

In order to enhance data classification and improve accuracy, electrical measurements from all 8 frequencies are combined and inputted into the machine learning algorithm. As evident from Fig. 8b, when compared to Fig. 8a, by integrating data from all 8 frequencies, there’s an approximate 8% improvement in categorization using solely electrical features. For test data based on optical features, there’s an improvement of around 15%. When both electrical and optical features are combined, there’s an approximately 11% enhancement compared to the test data from a single representative frequency.

Table 3 presents the True Positive Rates (TPR) obtained for identifying and categorizing each particle class at representative frequency of 500 kHz. The integration of electrical and optical features yielded improvements in classification accuracy. In addition to accuracy and TPR, confusion matrices provide a valuable metric for evaluating the performance of machine learning algorithms (Beauxis-Aussalet and Hardman 2014; Marom et al. 2010).

**Table 3:** True positive rates for electrical, optical, and multimodal features in the test and train datasets at 500KHz Frequency

	TPR	Electrical	Optical	Multimodal
Class 1	Train%	82.6	97.7	98.5
	Test%	59.6	97.9	100
Class 2	Train%	31	83.1	77.5
	Test%	36.3	42.5	67.1
Class 3	Train%	93.7	96.2	96.2
	Test%	84	100	88
Class 4	Train%	81.1	97.3	97.3
	Test%	100	100	100

**Fig. 9:**
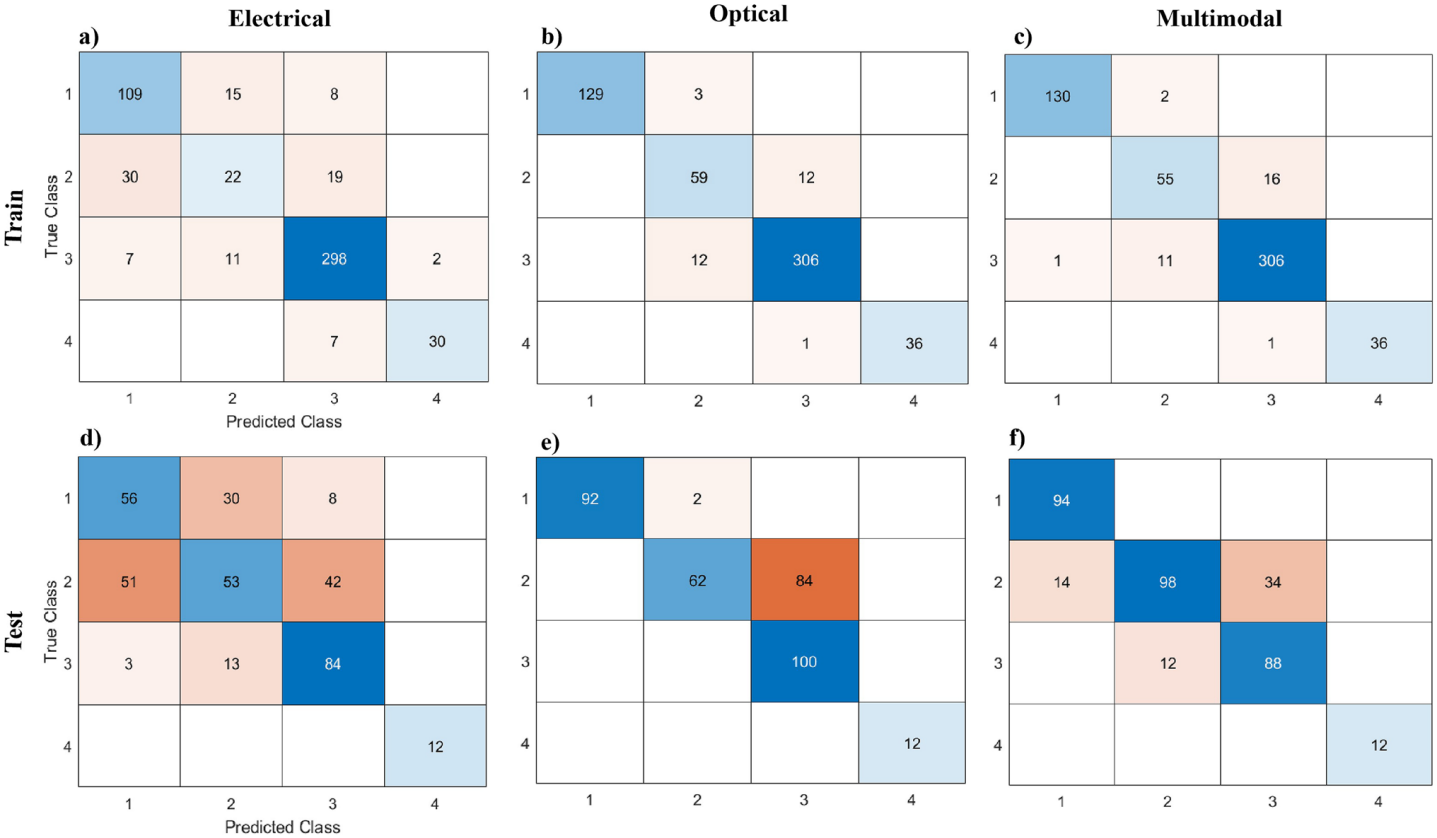
Confusion matrices obtained from the **a** electrical, **b** optical, and **c** multimodal classifiers on train dataset. Confusion matrices obtained from the **d** electrical, **e** optical, and **f** multimodal classifiers on test dataset

Figure 9 presents the confusion matrices obtained from the electrical, optical, and combined electrical and optical features on both the train and test datasets at frequency of 500 kHz. For instance, let us consider the classification of class 2, which corresponds to 4 μm beads. The confusion matrix reveals that when using electrical features on the test dataset, there were 51 particles erroneously classified as class 1 (2.8 μm bead size) and 42 particles misclassified as class 3 (5 μm bead size). However, upon incorporating the optical feature (volume feature), the classification improved significantly, eliminating misclassifications in class 1. Nonetheless, there were still 84 particles from class 2 that were misclassified as class 3. By incorporating both electrical and optical features, the classification accuracy is further enhanced, resulting in a reduction of misclassified particles from 84 to 48. This demonstrates the effectiveness of utilizing a combination of features in improving the precision of particle classification.

The utilization of confusion matrices allows for a more comprehensive evaluation of the machine learning algorithm’s performance, providing insights into the specific misclassifications made for each particle class. These findings highlight the effectiveness of integrating optical features, alongside electrical features, to enhance the classification accuracy, particularly when dealing with closely related particle sizes.

Table 4 provides a visual representation of the True Positive Rate (TPR) values obtained from both the training and test datasets, employing a comprehensive approach that involves combining data from all available frequencies. This strategic integration of data from all available frequencies has a significant impact on the accuracy of the classification model. By incorporating information from various frequencies, the model becomes capable of capturing a wider range of features and nuances present within the data. When using data from just one frequency, the model’s capacity to distinguish between various categories can be limited. Some patterns may be clearer at particular frequencies, while others might be less evident. By including data from all frequencies, the model gains a comprehensive view of the information, resulting in more accurate and dependable predictions. The increase in the TPR values underscores the advantages of this method.

**Table 4:** True positive rates for electrical, optical, and multimodal features in the test and train datasets at 500 kHz frequency

	TPR	Electrical	Optical	Multimodal
Class 1	Train%	71	100	99.2
	Test%	51.8	97.9	98.5
Class 2	Train%	49.8	96.8	97.6
	Test%	55.6	97.5	83.8
Class 3	Train%	96.5	97.3	98.4
	Test%	82.1	81.6	96.5
Class 4	Train%	87.5	100	99.7
	Test%	93.1	87.5	87.5

### Performance comparison

To compare our methodology with prior research that employed electrical, optical, and combined features for particle classification, we conducted a comparison with the work of D’Orazio et al. (2021). In their study, they proposed a novel multimodal approach that integrates electrical sensing and optical imaging to classify pollen grains flowing in a microfluidic chip, achieving a throughput of 150 grains per second. They reported accuracy values of 82.8% for the electrical classifier, 84.1% for the optical classifier, and 88.3% for the multimodal classifier on their training dataset. It is important to acknowledge that they also observed improvements when employing a multimodal approach. Their research reported an accuracy of 88.3% for the multimodal classifier on the training dataset, indicating the potential benefits of combining electrical and optical features in particle classification. Our study builds upon this valuable insight and further advances the multimodal approach. By integrating electrical and optical features, our methodology achieves a notably higher accuracy of 98.4% on training dataset. These results reinforce the notion that multimodal approaches have the potential to improve particle classification accuracy. Our study humbly contributes to this understanding by showcasing the effectiveness of our methodology in achieving higher accuracy through the combination of electrical and optical features.

## Conclusion

In this study, we investigated the integration of electrical and optical features through a multimodal approach for particle classification. Machine learning classifier algorithms were employed to evaluate the impact of combining these measurements. The results demonstrated that the multimodal approach yielded significantly improved accuracy compared to analyzing electrical or optical features independently. Electrical features achieved an average test accuracy of 66.4%, while optical features achieved 90.7%. However, by integrating both modalities, the average accuracy reached an impressive around 95%. This highlights the benefits of leveraging the complementary information provided by electrical and optical features in particle classification.

Our study underscores the importance and effectiveness of integrating electrical and optical features for particle classification. The multimodal approach outperformed individual modalities, offering a more robust and comprehensive solution. The findings demonstrate the unique strengths of each modality and emphasize the significance of their synergistic integration. This multimodal strategy has the potential to advance particle classification and analysis in various fields, providing deeper insights into cell properties and contributing to a better understanding of complex biological systems.

The integration of electrical and optical sensing modalities in our device facilitates the classification of particles in the range of 2.8 to 17 micrometers, crucial for healthcare diagnostics and therapeutics, despite challenges such as calibration complexity, resolution limits, and higher production costs. Precise classification, including macrovesicles (0.1–1.0 μm) and apoptotic bodies (1–5 μm), holds significant promise for early disease detection by identifying biomarkers for conditions like cancer and cardiovascular diseases, and in the drug delivery field for ensuring optimal nanoparticle carrier size distribution. Overcoming technical demands through advanced algorithms and diverse dataset collection is essential for enhancing the device’s reliability, generalizability, and affordability, aiming to revolutionize healthcare diagnostics and therapeutic delivery with minimal side effects.

Moving forward, future research can focus on several aspects to further enhance multimodal particle classification. Firstly, utilizing a camera with higher resolution can enhance the accuracy of volume estimation and reduce errors when obtaining particle volume from optical measurements. This improvement in image quality can contribute to more precise and reliable characterization of particles. Overall, future work in this field should aim to leverage technological advancements, expand the dataset diversity, and explore novel algorithms to further enhance the performance and applicability of multimodal particle classification systems. By addressing these aspects, we can unlock new possibilities for accurate and comprehensive particle analysis in various domains.

## Data Availability

Data is provided within the manuscript.
